# Intranasal Delivery of Nerve Growth Factor in Neurodegenerative Diseases and Neurotrauma

**DOI:** 10.3389/fphar.2021.754502

**Published:** 2021-11-16

**Authors:** Luigi Manni, Giorgio Conti, Antonio Chiaretti, Marzia Soligo

**Affiliations:** ^1^ Institute of Translational Pharmacology, National Research Council of Italy (CNR), Rome, Italy; ^2^ Department of Emergency, Intensive Pediatric Therapy and Pediatric Trauma Center, Anesthesiological and Reanimation Sciences, Fondazione Policlinico Universitario A. Gemelli IRCCS, Rome, Italy; ^3^ Department of Woman and Child Health, Institute of Pediatrics, Fondazione Policlinico Universitario A. Gemelli IRCCS, Rome, Italy

**Keywords:** nerve growth factor, intranasal delivery, pharmacology, neurodegeneration, neurotrauma and neurodegenerative disease

## Abstract

Since the 1980s, the development of a pharmacology based on nerve growth factor (NGF) has been postulated for the therapy of Alzheimer’s disease (AD). This hypothesis was based on the rescuing effect of the neurotrophin on the cholinergic phenotype of the basal forebrain neurons, primarily compromised during the development of AD. Subsequently, the use of NGF was put forward to treat a broader spectrum of neurological conditions affecting the central nervous system, such as Parkinson’s disease, degenerative retinopathies, severe brain traumas and neurodevelopmental dysfunctions. While supported by solid rational assumptions, the progress of a pharmacology founded on these hypotheses has been hampered by the difficulty of conveying NGF towards the brain parenchyma without resorting to invasive and risky delivery methods. At the end of the last century, it was shown that NGF administered intranasally to the olfactory epithelium was able to spread into the brain parenchyma. Notably, after such delivery, pharmacologically relevant concentration of exogenous NGF was found in brain areas located at considerable distances from the injection site along the rostral-caudal axis. These observations paved the way for preclinical characterization and clinical trials on the efficacy of intranasal NGF for the treatment of neurodegenerative diseases and of the consequences of brain trauma. In this review, a summary of the preclinical and clinical studies published to date will be attempted, as well as a discussion about the mechanisms underlying the efficacy and the possible development of the pharmacology based on intranasal conveyance of NGF to the brain.

## Introduction

The physiological peculiarity of the nerve growth factor (NGF) to regulate the survival and phenotype maintenance of specific neuronal populations in the peripheral and central nervous system (PNS and CNS, respectively) has laid the foundation for a broad line of preclinical and clinical research, aimed at exploring its pharmacological potential for the treatment of neurodegenerative diseases and of the outcomes of neurotrauma (Aloe et al., 2012; Allen et al., 2013). The enormous amount of preclinical research, conducted on a large number of *in vitro* and *in vivo* models, has indicated Alzheimer’s disease (AD) as a primary field of intervention (Cattaneo et al., 2008; Cattaneo and Calissano, 2012). The rationale for this therapeutic approach stems from the selective effect of NGF on the basal forebrain cholinergic neurons (BFCNs) (Hefti et al., 1984; Hefti, 1986) and from the evidence that the circuits connecting BFCNs to the cortex and hippocampus undergo early suffering during the development of AD (Whitehouse et al., 1981; Bartus et al., 1982). This rationale has subsequently been expanded by the accumulation of evidence regarding non cholinergic-specific actions exerted by NGF (Chiaretti et al., 2008; Calissano et al., 2010; Cragnolini et al., 2018; Rizzi et al., 2018). Furthermore, preclinical and clinical data on the pharmacological efficacy of NGF, indicated that this was severely limited by poor permeability of the molecule to the blood-brain barrier (Poduslo and Curran, 1996; Thorne and Frey, 2001) and by the possibility that side effects such as hyperalgesia, myalgias and weight loss, could outweigh the therapeutic benefits (Aloe et al., 2012). This brief review will focus mainly on the clinical experience gained to date, regarding the administration of NGF to the brain of patients suffering from neurodegenerative diseases and from the outcomes of neurotrauma. For a more in-depth discussion of the preclinical studies that have supported the clinical trials conducted so far, the reader is referred to more extensive reviews (Colafrancesco and Villoslada, 2011; Aloe et al., 2012; Cuello et al., 2019; Mitra et al., 2019).

## An extended rationale for the use of NGF in diseases of the central nervous system

NGF is the first discovered growth factor and a member of the neurotrophin family (Levi-Montalcini, 1952, 1987). It is synthesized as a pro-peptide (proNGF) starting from two splicing variants currently identified in humans (Scott et al., 1983; Ullrich et al., 1983; Edwards et al., 1986; Soligo et al., 2020a). The intracellular and/or extracellular processing of proNGFs generates a C-terminal mature fragment of 118–120 aminoacids (Seidah et al., 1996; Bruno and Cuello, 2006), which is the molecule currently under investigation for its pharmacological potential. NGF activates the tropomyosin receptor kinase A (TrkA) (Klein et al., 1991) and/or the p75 pan-neurotrophin receptor (p75NTR) (Johnson et al., 1986). The interaction between the two receptors, whether or not associated in hetero-complex, greatly increases the affinity (kd = 0.03 nM) for the binding of NGF to TrkA (Barker, 2007; Wehrman et al., 2007).

In the CNS, NGF is primarily neurotrophic for cholinergic neurons of the basal forebrain (Hefti, 1986) and for both healthy developing and damaged adult cholinergic interneurons in the striatum (Gage et al., 1989). During adult life, NGF, produced by BFCN-targets of innervation (Korsching et al., 1985), controls the maintenance of the cholinergic phenotype regulating the expression of choline-acetyltransferase (ChAT) (Gnahn et al., 1983; Pongrac and Rylett, 1998). The synthesis and release of NGF could be in turn regulated by the cholinergic activity and the release of acetylcholine (Knipper et al., 1994; Bruno and Cuello, 2006). Once released, NGF is internalized by the cholinergic endings and retrograde transported to the neuronal Soma (Seiler and Schwab, 1984). Thus, the canonical rationale for the treatment of AD patients with NGF is based on reported defective retrograde transport of NGF toward BFCN (Mufson et al., 1995) and on the accumulation of proNGF, that may have neurotoxic action (Lee et al., 2001), in the brain of AD patients (Fahnestock et al., 2001).

**FIGURE 1:**
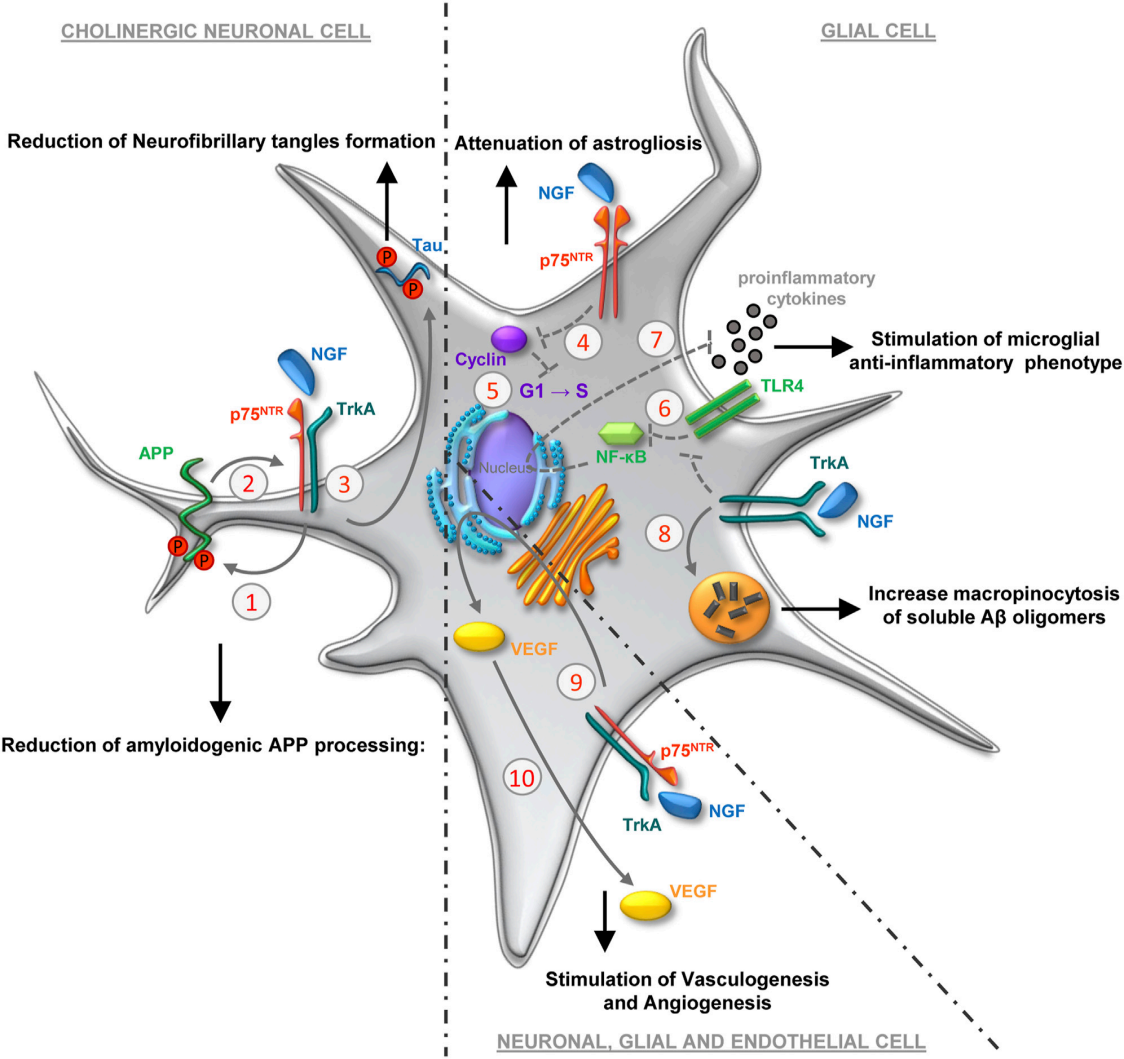
Molecular and cellular mechanisms underlying the therapeutic effect of NGF in the central nervous system. Other than the canonical effects on the phenotypic maintenance of basal forebrain cholinergic neurons, several mechanisms have been proposed to explain the outcomes elicited by the conveyance of exogenous NGF to the brain. The phosphorylation of APP (1) is regulated by the rate of APP/NGF-receptor association (2), in turn modulated by the interaction of NGF with the homodimer TrkA/TrkA and/or heterodimer TrkA/p75NTR (Canu et al., 2017b). The APP/NGF-receptor association makes APP less prone to be processed by β- and γ-secretases, resulting in decreased Aβ oligomerization in neurons expressing NGF receptors. The binding of NGF to its receptor complex reduces tau neurofibrillary tangles formation (3), regulating the post-translational modification of tau (phosphorylation, cleavage, and ubiquitination) (Canu et al., 2017b). In astrocytes, NGF/p75NTR interaction attenuates the induction of cyclins (4), thereby promoting the withdrawal of astrocytes from the cell cycle (5) attenuating astrogliosis (Cragnolini et al., 2012). NGF, inhibiting early TLR4-mediated activation of the NFκB (6) and JNK pathways, attenuates pro-inflammatory cytokines release in microglia (7) and may thereby contribute to regulation of microglia-mediated neuroinflammation (Fodelianaki et al., 2019). NGF-TrkA binding modulates microglia motility, macropinocytosis and degradation of Aβ deposition (8) (Rizzi et al., 2018). NGF initiates signaling (9) that supports the production and release of VEGF (10), in turn involved in both vasculogenesis and angiogenesis (Samii et al., 1999).

Preclinical and clinical studies have also demonstrated a pharmacological value of NGF in the treatment of neurotrauma outcomes (Kromer, 1987; Cacialli, 2021). The rationale behind these studies does not necessarily include the effect of NGF on cholinergic neurons, but extends to other peculiarities of the biological action of NGF. An extension of the therapeutic mechanisms triggered by NGF has been proposed based on the relationship between NGF, its receptors and the metabolism of the amyloid precursor protein (APP) and the protein tau (Cattaneo et al., 2008) (Figure 1). Altered metabolism of APP and tau are reported in a wide spectrum of neurological diseases (Gasparini et al., 2007; Hellewell et al., 2010; Zhang et al., 2018; Lim et al., 2019; Edwards et al., 2020). Described hallmarks of both neurodegenerative diseases and neurotraumas are altered processing of APP, the formation of 40–42 aminoacids-long peptides (amyloid-β: Aβ-40, Aβ-42) and their aggregation in the β-amyloid plaques, as well as the excessive phosphorylation and truncation of the tau protein, its aggregation and loss of function as a stabilizer of microtubules (Walsh and Selkoe, 2004; Gong and Iqbal, 2008; Xu et al., 2021). A direct interaction between APP and TrkA has been demonstrated, which, if disturbed by the presence of the Aβ peptides, is correlated to the induction of apoptosis (Canu et al., 2017a; 2017b). Furthermore, NGF binding to TrkA may route the APP metabolism toward the non-amyloidogenic processing, by modulating the interaction of APP with secretases (Canu et al., 2017a; 2017b). It is known that the amyloidogenic cascade is activated following NGF deprivation (Capsoni et al., 2000; Matrone et al., 2008; Latina et al., 2017) and in transgenic mice overexpressing proNGF (Tiveron et al., 2013). Such deprivation of NGF and/or increased proNGF/NGF ratio, both *in vitro* and *in vivo*, also leads to increased phosphorylation of tau and its abnormal cleavage (Nuydens et al., 1997; Capsoni et al., 2000; Shen et al., 2018; Mufson et al., 2019). Overall, these evidences suggest that NGF-based therapy could improve neurological outcomes that are related to dysfunctions of the central cholinergic system, both in neurodegenerative diseases (Mufson et al., 2019) and after TBI (Shin and Dixon, 2015), normalizing APP and tau metabolism in TrkA-expressing cells.

NGF regulates the functions of astrocytes and microglia (Pöyhönen et al., 2019) (Figure 1), modulating the glial response especially in conditions of suffering and/or trauma of the nervous system. NGF may modulate astrogliosis by arresting the cell cycle of astrocytes (Cragnolini et al., 2012). It may also act in anti-amyloidogenic way by regulating the inflammatory response of microglia (Capsoni et al., 2017; Rizzi et al., 2018) and decreasing the pro-inflammatory response through the reduction of microglial glycolysis (Fodelianaki et al., 2019). Moreover, NGF treatment leads to a modulation of microglia motility, micropinocytosis and degradation of Aβ deposition (Rizzi et al., 2018). This may account for non-TrkA-mediated, indirect action of NGF on the clearance of oligomers and aggregates in the brain of AD or TBI patients. The description of the complex glial function during neurological diseases goes beyond the scope of this work, (for recent reviews on the topic see: Rasband, 2016; Meyer and Kaspar, 2017; Stevenson et al., 2020). Nevertheless, it is important to underline that through its modulation of glial function, NGF may promote the establishment of a *milieu* advantageous to the processes of neuroprotection and neurorepair.

Strengthening this last consideration, is the positive effect on brain perfusion observed after administration of NGF to the brain of both laboratory animals and humans (Raychaudhuri et al., 2001; Cantarella et al., 2002; Dolle et al., 2005; Chiaretti et al., 2008; Jadhao et al., 2012). NGF has a pro-angiogenic activity. Inducing the production of vascular-endothelial growth factor (VEGF) (Samii et al., 1999; Graiani et al., 2004; Manni et al., 2005), a growth factor expressed either by neurons, glia and endothelial cells (Ogunshola et al., 2000; Nag et al., 2002), NGF may promote the proliferation and migration of endothelial cells (Chiaretti et al., 2002; Emanueli et al., 2002; Graiani et al., 2004; Salis et al., 2004) (Figure 1). Moreover, NGF stimulates the production of vasodilating agents, such as nitric oxide (Nizari et al., 2021). Furthermore, intranasal NGF is able to stimulate neo-angiogenesis following cerebral infarction in rats by activating PI3k/Akt signaling (Li et al., 2018). Overall, these mechanisms may underlie the observed increase in brain perfusion after NGF delivery to the human brain (Olson et al., 1992; Eriksdotter-Jönhagen et al., 1998; Tuszynski et al., 2005; Chiaretti et al., 2008, 2017, 2020; Fantacci et al., 2013; Rafii et al., 2018). Finally, the indirect action exerted by NGF on cerebral perfusion by stimulating the innervation of the cerebral vasculature (Isaacson et al., 1990) and the possible role of NGF-modulated glial regulation of brain perfusion and metabolism (Rasband, 2016), should not be underestimated.

### Intraparenchymal and Intracerebroventricular Delivery of NGF to the Human Brain

Since 1991, the administration of NGF to the human brain has been pursued through delivery to the brain parenchyma (intraparenchyma: IP) or cerebral ventricles (intracerebroventricular: ICV). The rationale was based on the action of NGF on NGF-responsive cells, therefore on BFCN in AD patients (Olson et al., 1992; Eriksdotter-Jönhagen et al., 1998, 2012; Tuszynski et al., 2005; Bishop et al., 2008; Rafii et al., 2014; Karami et al., 2015; Eyjolfsdottir et al., 2016; Machado et al., 2020) or on catecholaminergic cells of adrenal origin transplanted into the brain of Parkinson’s patients (Olson et al., 1991). Only in some compassionate studies NGF has been administered ICV to pediatric patients suffering from severe hypoxic-ischemic trauma, not aiming at stimulating selectively the cholinergic function (Chiaretti et al., 2005, 2008; Fantacci et al., 2013). The delivery systems, whether purified NGF was delivered, whether it was the inoculation of adenovirus for gene therapy or those of NGF-producing cells, involved invasive, relatively risky surgical procedures for administration/implantation, difficult to configure in view of the need for large-scale treatments. For detailed description and methodological consideration about the delivery of NGF to CNS in the above-cited clinical studies the reader is referred to more comprehensive reviews (Thorne and Frey, 2001; Aloe et al., 2012; Wahlberg et al., 2012; Mitra et al., 2019; Eftimiadi et al., 2021).

Here, it is important to underline some aspects that integrate the clinical experiences related to the delivery of NGF to the brain, so far reported in clinical trials (some of which are accessible on https://clinicaltrials.gov/: NCT00017940, NCT01163825, NCT00087789, NCT00876863). In AD patients, most studies reported increased activity of nicotinic receptors, measured through the incorporation of ^11^C-nicotine (Olson et al., 1992; Eriksdotter-Jönhagen et al., 1998, 2012; Tuszynski et al., 2005; Karami et al., 2015). The data from a specific trial (NCT01163825) also highlighted, albeit only on patients defined as “responders”, increase in the activity of ChAT and AChE in the CSF following delivery of NGF mediated by cellular implants in the basal forebrain. These outcomes showed positive correlation with a slower cognitive decline, increased glucose uptake, increased ^11^C-nicotine binding, decreased Aβ-42 and phospho-tau levels in CSF (Eriksdotter-Jönhagen et al., 2012; Karami et al., 2015; Eyjolfsdottir et al., 2016; Mitra et al., 2019). The assessments of cognitive status, although not consistently showing effects related to NGF treatments, indicate the possibility of slowing the progression of AD through the supplementation of NGF to BFCNs (Tuszynski et al., 2005; Eyjolfsdottir et al., 2016). The improvement in the fast-to-slow waves ratio recorded in the EEG in several of the studies mentioned so far, also indicates the potential efficacy of NGF in correcting neurophysiological deficits observed in AD patients.

One of the most relevant and common effects of NGF in the aforementioned studies, was the increase in cerebral perfusion and ^18^F-FDG uptake, an index of increased glucose metabolism found in various brain areas. The mechanisms underlying the effect of NGF on cerebral perfusion have already been addressed above. The augmented metabolism could be linked to the increased availability of nutrients, the rise in the septum-cortical circuits activity (Tuszynski et al., 2005) or also to specific effects of NGF on the metabolism of NGF-responsive neurons and glia (Rasband, 2016; Colardo et al., 2021).

As for the development of side effects, in the clinical studies conducted so far (Eriksdotter-Jönhagen et al., 1998) they have been mainly related to the insurgence of hyperalgesia and allodynia (back pain, myalgia) and to the onset of an anorectic effect (Lapchak and Araujo, 1994) with consequent weight loss. It should be noted that these effects are generally reversible and dose-dependent (Eriksdotter-Jönhagen et al., 1998) and that they occurred following ICV, but not after IP delivery.

Although characterized by encouraging indication about the safety and tolerability of some of the procedures used to deliver NGF to the brain parenchyma (Tuszynski et al., 2005; Eriksdotter-Jönhagen et al., 2012; Rafii et al., 2014; Eyjolfsdottir et al., 2016), these studies have not yet laid the foundation for the development of a NGF pharmacology based on invasive neurosurgical procedures. Indeed, a recent post-mortem study revealed the failure of targeting BFCN after virus-mediated NGF gene delivery, due to the limited spread of the vector from the injection site (Castle et al., 2020). On the other hand, the encapsulated cells biodelivery of NGF appears to be in an early stage of development, still being hampered by variations in the levels of NGF-release between implants, inconsistent cells viability and inflammatory reactions due to surgical procedures (Mitra et al., 2019).

### Intranasal Delivery of Nerve Growth Factor to the Brain

The non-invasive, intranasal delivery of biomolecules, aimed at bypassing the blood-brain barrier and reaching the brain parenchyma, has been extensively explored since the end of the last century (Frey et al., 1995, 1997; Thorne et al., 1995; Chen et al., 1998) and several patents by Frey et al. claim intranasal delivery of drugs to the brain along the olfactory neural pathway (Frey, 1997), and the trigeminal neural pathway (Jogani et al., 2008).

**FIGURE 2:**
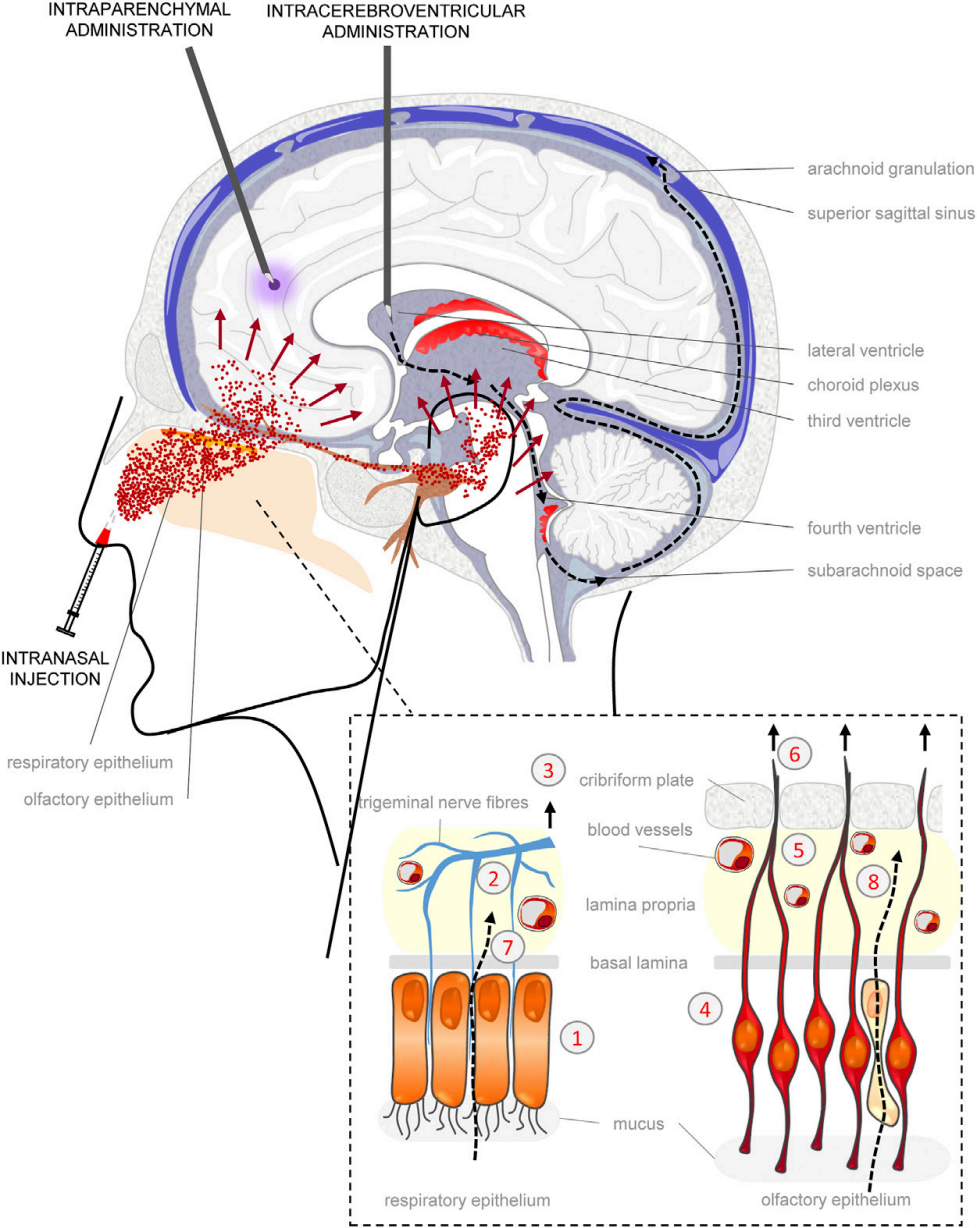
Mechanisms of exogenous biomolecule distribution to the brain tissue. Intraparenchymal (IP) administration allows the local delivery of biomolecules in CNS tissue, limiting distribution to an area no more than about 2 mm from the site of introduction (violet spread). Intracerebroventricular (ICV) administration may deliver biomolecules to wide areas of CNS as a result of circulation within cerebrospinal fluid (CSF) (black dotted arrows). From the lateral ventricle, through the fourth ventricle biomolecules can reach the subarachnoid space where the CSF is filtered by arachnoid granulations in the bloodstream of the superior sagittal sinus. However, many limitations affect ICV administration: penetration into the underlying parenchymal tissue (about 2 mm), rapid clearance (NGF half-life < 1 h, in the 150 ml of CSF, which is replaced entirely within 8 h), sequestration because of binding to protein component of CSF. Intranasal (IN) injection is a non-invasive alternative to both IP and ICV administrations that permits direct delivery to CNS bypassing the BBB (Lochhead and Thorne, 2012). Nose-to-brain passage of biomolecules (red spray) may occur either by intracellular or extracellular pathways both in the respiratory and olfactory epithelium (enlarged box at the bottom right), allowing drugs to reach in almost every brain region. The intracellular transport occurs through: endocytosis across the respiratory epithelium (1), toward the peripheral trigeminal nerve (2) and transport to brainstem (3); endocytosis into olfactory sensory neurons (OSN) (4) that extend across the basal lamina and converge with axons from other OSN to form nerve bundles (5) projecting to the olfactory bulbs, piriform cortex, amygdala and entorhinal cortex (6); transcytosis to the lamina propria across other cells of the respiratory epithelium (7) and sustentacular cells (8) of olfactory epithelium (Lochhead and Thorne, 2012). The extracellular pathways consist of paracellular diffusion within perineural, perivascular or lymphatic channels associated with trigeminal and olfactory fibres that enter the brain (Lochhead and Thorne, 2012), and is the preferential route of diffusion of NGF into the brain, due to the lack of TrkA expression on the olfactory epithelium that limit the intracellular entry and transport (Frey et al., 1997).

The transport of drugs from nose to brain occurs after conveyance to the olfactory epithelium, the uppermost part of the nasal cavity that contains the olfactory sensory neurons (Dhuria et al., 2010; Lochhead and Davis, 2019). The transport to the brain (Figure 2) can occur by extracellular and intracellular pathways and through diffusion in the perivascular and perineural spaces of the olfactory and trigeminal nerves (Dhuria et al., 2010; Lochhead and Davis, 2019). Once it reaches the brain, rostrally via the olfactory pathways and caudally via the trigeminal nerve, the drug rapidly diffuses into the cerebral perivascular spaces, potentially distributing itself throughout the whole cerebral parenchyma (Lochhead and Davis, 2019).

Intranasally-delivered iodinated NGF (IN-NGF), unlike NGF administered intravenously, was able to rapidly (within 20 min from inoculation) spread in the brain (Frey et al., 1997). IN-NGF was mainly found in the olfactory bulbs and the brainstem, albeit distributed in the whole brain region between them. (Frey et al., 1997). Of note, only 0.3% of exogenous NGF was found in the bloodstream after IN-NGF (Frey et al., 1997). These results, obtained after delivery of iodinated NGF, were confirmed by subsequent studies, in which native murine NGF (Chen et al., 1998) or a “painless” human NGF mutein (Capsoni et al., 2017) were IN delivered and detected by ELISA. Based on these findings, on the linear relationship between the intranasal dose and resulting brain concentration and on the known absence of TrkA receptors on the olfactory epithelium, an extracellular and perineural/perivascular pathway of diffusion was hypothesized (Frey et al., 1997; Chen et al., 1998). Interestingly, despite diffusion of IN-NGF in the CSF was predictable, due to the connection between perineural and perivascular spaces and nasal lymphatics with the subarachnoid space (Dhuria et al., 2010) (Figure 2), low levels of IN-NGF were found in the CSF by ELISA (Chen et al., 1998).

IN-NGF has been extensively studied in preclinical models of AD, using NGF brain-deprived mice (AD11 mice), or multiple-transgenic models, co-expressing mutated forms of APP and presenilin 1 (APPxPS1) or comprising five familial Alzheimer’s disease mutations (5xFAD). IN-NGF, delivered in its native form or as a “painless” mutein, improved neurodegenerative symptoms (Capsoni et al., 2002) by ameliorating cholinergic deficits (Covaceuszach et al., 2009; Capsoni et al., 2012), decreasing tau phosphorylation (Capsoni et al., 2009, 2012; Covaceuszach et al., 2009), APP metabolism and Aβ plaque deposition (Covaceuszach et al., 2009; Capsoni et al., 2012, 2017; Yang et al., 2014), at the same time rescuing both recognition-spatial memory deficits (De Rosa et al., 2005; Capsoni et al., 2012, 2017), hippocampal and LTP deficits (Capsoni et al., 2017). IN-NGF also counteracted microglia and astrocytes activation, Aβ presence in both cellular types and the production of pro-inflammatory cytokines (Capsoni et al., 2012, 2017).

Other relevant models of neurological pathologies in which the efficacy of IN-NGF has been attempted, include cerebral ischemia, traumatic lesions of the brain and spinal cord, epilepsy, amyotrophic lateral sclerosis, hypogonadism related to premature aging, and depression. Also in these models, IN-NGF improved selective behavioral performances (Cheng et al., 2009; Shi et al., 2010; Bianchi et al., 2012; Tian et al., 2012; Aloe et al., 2014; Zhong et al., 2017), Aβ plaque deposition and tau phosphorylation (Tian et al., 2012; Lv et al., 2014), and promoted anti-inflammatory response (Lv et al., 2013). It also decreased seizure onset (Lei et al., 2017), counteracted disease-induced apoptosis (Cheng et al., 2009; Lv et al., 2013; Lei et al., 2017), enhanced VEGF and endothelial cell migration (Li et al., 2018), enhanced neurogenesis (Cheng et al., 2009; Zhu et al., 2011), regulated hypothalamic gonadotropin releasing hormone production (Luo et al., 2018). Only one study found IN-NGF not effective in ameliorating motor functions impaired by brain trauma (Young et al., 2015).

A total number of four patients have so far been treated with IN-NGF. In the first case-report (Chiaretti et al., 2017) murine NGF was intranasally delivered in a 4-years-old boy suffering for the consequences of a severe TBI. The patient received one cycle (100 μg/kg twice a day for 10 consecutive days) each month for 4 months of IN-NGF. This regimen progressively improved brain perfusion and brain metabolism, increased EEG fast/slow waves ratio, reduced ventricular dilatation and parenchymal lesions and normalized the size of subarachnoid spaces. No side effects related to NGF therapy were reported, related to nociceptive hyper-response or autonomic abnormalities, despite a modest increase of NGF content in the CSF. A second clinical study (de Bellis et al., 2018) reported IN-NGF in two adult patients affected by frontotemporal dementia associated with corticobasal syndrome. Patients received 2 µg/day of murine NGF for a 1-year period. A dose escalation to 4 and 6 µg/day was attempted and the insurgence of reversible side effects (rhinitis, rigidity, moderate psychomotor agitation) recorded. Significant reduction in the mini-mental state examination score was observed and returned to pre-treatment conditions within 1 year after stopping NGF treatment. PET-scans revealed a progressive and significant increase in FDG-uptake in several cortical and subcortical brain areas, which was also reverted to pre-treatment levels after IN-NGF interruption. In a third case-report (Chiaretti et al., 2020) a 7-weeks-old infant with persistent wakefulness syndrome due to late-onset group-B *Streptococcus* meningitis was treated with commercial human recombinant NGF (Oxervate^®^, Dompè Farmaceutici). The infant received five monthly cycles of intranasal NGF (20 µg/day for seven consecutive days). IN-NGF promoted a progressive improvement of brain hypometabolism, increasing glucose uptake in cortical and subcortical regions. Clinical scales for assessment of comatose and cognitive states all improved after the study protocol was completed.

A EU-registered therapeutic exploratory (phase II) clinical trial (https://www.clinicaltrialsregister.eu/ctr-search/trial/2019-002282-35/IT) on five children aged between 6 months and 5 years, and affected by severe neurosensory, cognitive and motor deficits after traumatic brain injury, is actually ongoing, aiming at producing evidence of changes in clinical and neurological conditions after treatment with 50 μg/kg of IN- rhNGF (Oxervate^®^, Dompè Farmaceutici).

## Discussion

The pharmacology of IN-NGF seems to be heading towards promising development, based on the ease of administration, the efficiency of drug distribution to the brain parenchyma and the efficacy demonstrated in a number of preclinical studies. Some points, in addition to those already discussed, deserve to be deepened, such as the role of exogenous NGF in modifying the proNGF/NGF ratio in the brain, the possible synergistic effects of other therapies to be associated with IN-NGF, the potential development of side effects and the development of proper IN delivery devices/strategies. Furthermore, some considerations should be made regarding the strategies for future research aimed at optimizing treatments protocols for IN-NGF, to be translated into clinical practice.

The delivery of NGF to the brain may change the balance between endogenous proNGF and mature NGF (mNGF), which if shifted toward the former, can itself promote the development of functional dysfunctions and neurodegenerative events. ProNGF is the prevalent form of NGF in the brains of AD patients (Fahnestock et al., 2001) and its processing into mature NGF may be impaired in neurological diseases (Cuello et al., 2010). The biological effect of proNGF and NGF may be opposite (Hempstead, 2014), especially when the neuronal distress increases p75NTR/TrkA ratio (Chakravarthy et al., 2012), favoring the binding of proNGF to p75NTR and the activation of the apoptotic cascade (Hempstead, 2014). Therefore, further investigation of these mechanisms after IN-NGF deserves attention and future work.

By being a facilitator of metabolism and perfusion, IN-NGF may impact a broad neuro-pathological spectrum. It is worth noting that, similarly to IN-NGF, intranasal insulin was able to enhance brain energy levels, to improve memory loss and to reduce white matter degeneration in MCI and AD patients (Craft et al., 2012; Jauch-Chara et al., 2012; Kellar et al., 2021). A synergistic combination of these intranasal growth factors may deserve, therefore, a specific investigation. The possible recovery of the physiological phenotype promoted by NGF in neurons and glia produced functional improvements in patients with established deficits and disabilities, but has been proven reversible (de Bellis et al., 2018). Therefore, it might be useful that IN-NGF be associated with physical therapies (e.g., transcranial direct current stimulation, vagal stimulation, electroacupuncture, physiotherapy) (Cheng et al., 2009) or stem cell transplantation (Zhong et al., 2017; Wang et al., 2020). These may selectively stimulate the recovery of the connectivity and plasticity of the damaged areas, being synergic in their action with the effects of IN-NGF and irreversibly consolidating the functional changes promoted by IN-NGF alone.

Until now, the pharmacology of NGF has been severely limited by the onset of side effects after systemic (Apfel, 2002) or intracerebroventricular (Eriksdotter-Jönhagen et al., 1998) delivery and by the difficulty of identifying a “therapeutic window” in which the therapeutic target is reached, maximizing the efficacy and minimizing or avoiding altogether the onset of adverse events (Cattaneo et al., 2008; Aloe et al., 2012). In the preclinical studies mentioned above, IN-NGF dosages and duration of administration were very heterogeneous. Furthermore, only in few cases were assessments on the safety of the treatment carried out. In particular, it has been found that at least up to a dose of 0.48 μg/kg of IN-NGF delivered three times a week for 2 weeks, there were no physiological and molecular indications for the development of painful symptoms (Capsoni et al., 2009). In clinical studies, at much higher doses than this latter, no side effects were found, attributable to the action of NGF, after IN delivery in TBI children (Chiaretti et al., 2017, 2020). This aspect will be further and specifically investigated, as a secondary endpoint, in the ongoing clinical trial mentioned in a previous section (https://www.clinicaltrialsregister.eu/ctr-search/trial/2019-002282-35/IT). When delivered at low daily dosage for a long period of time in adult patients (de Bellis et al., 2018), any effects on nociceptive or autonomic systems have been recorded, while other reversible and dose-dependent side effects were noticed (rhinitis, rigidity, moderate psychomotor agitation). The possibility that IN-NGF may, at therapeutic doses, partly diffuse into the CSF (Thorne and Frey, 2001), sensitizing spinal neurons, cannot be ruled out (see Figure 2). However, one must take into account the short half-life of NGF (less than 1 h after ICV) (Lapchak et al., 1993), and that the percentage of IN-NGF spreading by perivascular and perineural space into the subarachnoid space (Dhuria et al., 2010) instead of in the parenchyma, may not be sufficient to reach the spinal cord neurons in relevant concentrations, especially after a delivery regimen limited to a few days. Nevertheless, in order to avoid potential development of side effects after IN-NGF, such as those related to pro-nociceptive function or loss of body weight, while inducing neurotrophic outcomes, the delivery of NGF-variants that target specifically the p75NTR (Manni et al., 2019; Soligo et al., 2019, 2020b) or that do not promote the phosphorylation of residue Tyr490 on TrkA, with subsequent activation of PLC-1 (Capsoni et al., 2011; Cattaneo and Capsoni, 2019), have been attempted. It should be noted, however, that these pharmacological approaches currently seem to be more suitable for systemic delivery of NGF, yet described as inducing side effects (Apfel, 2002).

Finally, much remains to be explored regarding the delivery technology. The physical and metabolic barriers that potentially hinder the penetration of IN-NGF into the cerebral parenchyma concern the anatomy of the human nasal cavity (Lochhead and Thorne, 2012; Gänger and Schindowski, 2018) and the rate of muco-ciliary clearance (Gänger and Schindowski, 2018). Regarding the latter, preclinical testing is underway on formulations that provide for the protection and increased absorption of NGF (Vaka et al., 2009; Vaka and Murthy, 2010; Luo et al., 2018), which can be obtained through lipid carriers, surfactants or polysaccharides (Erdő et al., 2018; Gänger and Schindowski, 2018). Also, the possibility exists of delivering NGF-mRNA through exosomes (Yang et al., 2020). As for physical obstacles to nose-to-brain delivery, the development of devices that maximize the deposition of drugs to the upper part of the nasal cavity, avoiding dispersion in the airways, or passage of the drug from the nasal mucosa to the blood circulation is underway but still not used to administer NGF (Djupesland et al., 2014; Gänger and Schindowski, 2018).
